# Latent profiles of 24-h movement behaviors and bone health at one-year follow-up in Chinese preschool children

**DOI:** 10.3389/fpsyg.2026.1964754

**Published:** 2026-10-13

**Authors:** Yuhan Li, Sen Cao

**Affiliations:** School of Education, Beijing Sport University, Beijing, China

**Keywords:** 24-h movement behaviors, bone health, compositional data analysis, latent profile analysis, preschool children, tibial speed of sound

## Abstract

**Background:**

Physical activity, sedentary behavior and sleep together occupy a fixed 24-h day, yet their relation to bone health in early childhood has largely been examined one behavior at a time, or through adherence to separate guideline thresholds. Person-centred approaches that characterise the whole-day composition have not been applied to bone health in preschool children.

**Methods:**

A total of 109 children (mean age 4.33 years; 44.0% girls) from three kindergartens in Changsha, China, were followed for 1 year. Sedentary behavior, light and moderate-to-vigorous physical activity were measured by hip-worn accelerometry over seven consecutive days, and sleep was reported daily by parents and recorded by class teachers including the weekend days. The four behaviors were closed to 1,440 min/day, expressed as isometric log-ratio coordinates, and summarised by latent profile analysis. Bone health was indexed by the tibial speed of sound (SOS) from quantitative ultrasound, expressed as a *z*-score for age, sex and ethnicity. Covariates were related to profile membership by the bias-adjusted three-step approach, and profiles to the SOS *z*-score by the Bolck–Croon–Hagenaars approach in baseline score and 1-year follow-up score models.

**Results:**

Three profiles were identified: Sedentary (*n* = 31), Low-active, long-sleeping (*n* = 7) and Active (*n* = 71). Older children were more likely to belong to the Active profile than to the Sedentary profile (OR 2.164 per year, 95% CI 1.012 to 4.630), whereas sex, socioeconomic status score and body mass index *z*-score were not associated with profile membership. At baseline the SOS *z*-score did not differ across profiles. At 1-year follow-up, children in the Active profile scored lower than those in the Sedentary profile (adjusted difference −0.462, 95% CI −0.774 to −0.150, *p* = 0.004, Holm-adjusted *p* = 0.016), whereas the Low-active, long-sleeping profile did not differ (−0.256, 95% CI −0.844 to 0.332).

**Conclusion:**

An association between 24-h movement profile membership and bone health was present at 1-year follow-up but not at baseline. The whole-day movement composition therefore merits consideration in bone health research and promotion in the preschool years.

## 1 Introduction

Bone mineral accrues throughout the growing years, and the amount attained by the end of skeletal growth defines peak bone mass; a one standard deviation higher peak bone mass is estimated to reduce the risk of osteoporotic fracture in adulthood by approximately 50% (Bonjour et al., 2009). Bone mineral density and bone mineral content increase during the preschool years (Specker et al., 2001; Hazell et al., 2015). At this age, bone health is associated with modifiable factors including physical activity, dietary intake, calcium and vitamin D status, and mechanical loading (Ekbote et al., 2011; Faienza et al., 2023). Identifying modifiable behaviors associated with bone health during the preschool years may therefore help inform early health promotion.

Among these modifiable factors, sedentary behavior (SB), physical activity, and sleep are each relevant to the developing skeleton. Weight-bearing and impact activity has been associated with greater bone mineral accrual in young children (Erlandson et al., 2011; Jackowski et al., 2015), although a meta-analysis found that exercise added beyond children’s usual activity did not improve densitometric measures of bone health, with the effect in preschool children largely unknown (McCaskie et al., 2023). SB has been associated with lower areal bone mineral density (Sioen et al., 2015), an association that weakens after accounting for moderate-to-vigorous physical activity (MVPA) (Herrmann et al., 2015). Longer sleep duration and daytime napping have been associated with higher bone mineral content (Casazza et al., 2011), and nocturnal sleep duration with greater bone strength in preschoolers (Fu et al., 2023). These behaviors have most often been examined in isolation, yet they are mutually exclusive parts of a fixed 24-h day, so that time in any one is time not spent in another.

This interdependence underpins the 24-h movement paradigm, in which the behaviors are considered together rather than in isolation (Tremblay et al., 2017), and it makes 24-h time-use data compositional, conveying relative rather than absolute information; compositional data analysis (CoDA) has been developed to model such data (Dumuid et al., 2020). In preschool children, compositional analyses have been related to adiposity (Fu et al., 2024), physical fitness (Song et al., 2024), and cognitive ability (Xu et al., 2025), but rarely to bone health. Bone health has instead been examined through adherence to separate guideline thresholds, which has been prospectively associated with higher bone mineral density in later childhood (Hinkley et al., 2020) and with a lower risk of low bone mineral density 1 year later (Li et al., 2024); the single compositional study to include bone in early childhood reported associations of small and clinically limited magnitude (Taylor et al., 2018). Most compositional analyses are variable-centred, estimating average associations or the effect of reallocating time across the sample, whereas person-centred approaches such as latent profile analysis identify subgroups of children who share qualitatively distinct 24-h patterns (Gupta et al., 2020); such typologies have been linked to adiposity in children and adolescents (Janda et al., 2024), and, recently, to physical fitness in preschool children (Yang et al., 2026), but not to bone health in this age group.

The present study aimed to identify person-centred profiles of 24-h movement behavior in preschool children using compositional latent profile analysis, and to examine their association with bone health, indexed by the tibial speed of sound (SOS), at baseline and 1 year later.

## 2 Methods

### 2.1 Study design and participants

This study is a secondary analysis of a prospective cohort of preschool children in which bone health was originally examined in relation to adherence to the 24-h movement guidelines (Li et al., 2024). The cohort’s baseline and 1-year follow-up records were reused to pursue a different aim: to identify person-centred profiles of 24-h movement composition and to examine their association with bone health at baseline and 1 year later.

Children were recruited from the junior and middle classes of three kindergartens in Changsha, China. These age groups were selected so that the same children could be reassessed 1 year later. Baseline measurements were conducted in the autumn of 2022. Of the 120 children initially enrolled, 8 were excluded at baseline (6 provided no valid accelerometer data and 2 no valid sleep data), leaving 112 children for longitudinal follow-up. One year later, 3 children were lost (2 withdrew and 1 transferred to another kindergarten), yielding a final analytic sample of 109 children with complete baseline and follow-up data.

The original study was conducted in accordance with the Declaration of Helsinki and approved by the Ethics Committee of Hunan Normal University (approval number 2022-334). Before testing, the purpose, procedures and possible inconveniences were explained to each child’s parent or guardian at a parents’ meeting, and written informed consent was obtained.

### 2.2 Measurement

#### 2.2.1 24-h movement behaviors

Physical activity and SB were assessed with a triaxial ActiGraph wGT3X-BT accelerometer (ActiGraph LLC, Pensacola, FL, USA). The device was fitted on the right hip with an elastic belt by trained researchers and worn for seven consecutive days (five school days and two weekend days), and was removed only for bathing and sleeping. Before fitting, each device was initialised in ActiLife software with the child’s sex, height, weight and date of birth, and data were collected in 15-s epochs. Non-wear time was identified with the Choi algorithm (Choi et al., 2011). A day was considered valid when wear time reached at least 10 h, and a child’s record was retained when at least three valid days, including at least one weekend day, were available. Time spent in SB, light physical activity (LPA) and MVPA was derived using the preschool intensity cut-points of Butte et al. (2014).

Sleep duration was obtained from a sleep diary. Parents reported each child’s bedtime and wake time daily over the seven consecutive days of accelerometer wear, including the weekend days. Class teachers collated the daily reports, and the research team cross-checked them against the accelerometer wear log. Average daily sleep duration, including daytime naps and night-time sleep, was calculated as a weighted mean of school-day and weekend-day values [(school-day sleep × 5) + (weekend-day sleep × 2)]/7.

#### 2.2.2 Bone health

Bone health was indexed by the SOS (m/s), an ultrasound measure of bone status in children (Foldes et al., 1995; Baroncelli, 2008). SOS was assessed by quantitative ultrasound using the Sunlight Omnisense 7000P device (Sunlight Medical, Israel), a non-invasive, radiation-free method, in triplicate at the midshaft of the non-dominant tibia, and expressed as a *z*-score, calculated as the difference between the measured value and the mean of children of the same age, sex and ethnicity, divided by the corresponding standard deviation (Zadik et al., 2003); a higher *z*-score therefore denotes a faster SOS along the tibial cortex than in the reference population.

#### 2.2.3 Other variables

Height and weight were measured at baseline, and body mass index (BMI) was calculated as weight divided by height squared. BMI-for-age *z*-scores (BMI-*z*) were derived from the WHO Child Growth Standards (WHO Multicentre Growth Reference Study Group, 2006; de Onis et al., 2007), and weight status was classified as underweight, normal, or overweight and obese using the corresponding age-specific cut-points. Family socioeconomic status (SES) was summarised from five indicators (paternal education, maternal education, paternal occupation, maternal occupation and household income); the first principal component of these indicators (explaining 52.7% of the variance) was retained as a continuous SES score and was additionally divided into tertiles. Age, sex, BMI-*z* and the continuous SES score were used as covariates in subsequent analyses.

### 2.3 Statistical analysis

The four behaviors (SB, LPA, MVPA and sleep) were treated as a four-part composition that carries only relative information (Aitchison, 1986; Dumuid et al., 2018). SB, LPA, and MVPA were derived from waking accelerometer wear and sleep from the diary, so that the four parts were mutually exclusive (Wu et al., 2023). For each child the four parts were closed to a constant sum of 1,440 min/day. Extreme values of each closed part were winsorised following Balbim et al. (2024), with observations beyond Q1 − 1.5 × IQR or Q3 + 1.5 × IQR replaced by the 5th and 95th percentiles, respectively. Because winsorisation breaks the constant-sum constraint, the composition was closed to 1,440 min/day a second time, so that the four parts sum to exactly 1,440 min/day for every child. The composition was then expressed as three isometric log-ratio (ilr) pivot coordinates from a sequential binary partition that contrasted MVPA against the geometric mean of the remaining behaviors, then SB against the geometric mean of LPA and sleep, and finally LPA against sleep.

Latent profiles were estimated from the three ilr coordinates by latent profile analysis, using a mixture model with 500 random start values (50 retained) (Janda et al., 2024; Yang et al., 2026). Within-class variances were freely estimated across profiles and within-class covariances were fixed at zero. Solutions from one to five profiles were compared on the Bayesian information criterion (BIC), the sample-size-adjusted BIC (aBIC), entropy, the Lo–Mendell–Rubin likelihood ratio test (LMR), the bootstrapped likelihood ratio test (BLRT), the size of the smallest profile, and substantive interpretability (Nylund et al., 2007; Yang et al., 2026). Each child was assigned to the profile with the highest posterior probability, and the composition of each profile was summarised by the compositional mean of the four behaviors (Gupta et al., 2020; Balbim et al., 2024). Classification quality was summarised by entropy and by the average posterior probability of the assigned profile. Profiles were named according to their SB, LPA, MVPA and sleep characteristics.

The association of each covariate (age, sex, BMI-*z* and the continuous SES score) with profile membership was examined using the bias-adjusted three-step (R3STEP) procedure, which regresses class membership on a covariate while accounting for classification uncertainty, with one profile specified as the reference category (Asparouhov and Muthén, 2014; Janda et al., 2024), each covariate being entered in a separate model. Missing values on a covariate are handled by listwise deletion in this procedure.

The association of profile membership with the tibial SOS *z*-score was estimated with the Bolck–Croon–Hagenaars (BCH) approach, in which each child is weighted by the inverse of the classification error matrix (Bolck et al., 2004; Asparouhov and Muthén, 2014). It was implemented as a manual two-step procedure in Mplus 8.3, because the built-in BCH auxiliary option does not allow covariates to be included in the outcome model. In the first step the three-profile model was re-estimated and the BCH weights were saved; each child is assigned the row of the inverted classification error matrix corresponding to the child’s most likely profile. In the second step these weights were supplied as training weights and the outcome model was fitted with the robust maximum likelihood estimator. Two models were fitted: the first with the baseline SOS *z*-score as the outcome, adjusted for age, sex, the continuous SES score and BMI-*z*; the second with the follow-up SOS *z*-score as the outcome, adjusted for the same covariates and for the baseline score. The covariates were entered with profile-specific means and with variances and covariances held equal across profiles, which prevents a singular within-profile covariance matrix in the smallest profile; regression slopes were also held equal across profiles, whereas the outcome intercept and residual variance were estimated separately for each profile. Because the covariates are part of this model, children with an incomplete covariate were retained by full information maximum likelihood. The two profile contrasts were defined as constraints on these intercepts, and their standard errors were obtained by the delta method. Associations were expressed as adjusted mean differences (*β*) in SOS *z*-score units with 95% confidence intervals (CI).

The same two contrasts were also estimated after assigning each child to the profile with the highest posterior probability, using HC3 heteroscedasticity-consistent standard errors; these models were fitted in R with listwise deletion. Within each specification, the two profile contrasts at each of the two timepoints were treated as a family and *p* values were adjusted by the Holm procedure. For each contrast, the smallest difference detectable with 80% power at a two-sided alpha of 0.05 was computed.

Sensitivity analyses comprised two- and four-profile solutions, related to the follow-up score by the same BCH procedure, and re-estimation of the association after excluding the smallest profile. The latent profile and R3STEP models were estimated in Mplus 8.3 with the robust maximum likelihood estimator, and all other analyses were conducted in R 4.4.2. The Mplus input files are provided in the Supplementary material.

## 3 Results

### 3.1 Sample characteristics

The 109 children were on average 4.33 (SD 0.61) years old at baseline, and 48 (44.0%) were girls (Table 1). Most were of normal weight (93.5%, mean BMI 15.04 kg/m^2^, BMI-*z* −0.27). Before closure the four behaviors summed to 1431.01 (36.71) min/day. After closure the composition averaged 493.15 (75.80) min/day of SB, 189.65 (39.17) min/day of LPA, 46.62 (19.53) min/day of MVPA and 710.58 (90.93) min/day of sleep. The mean tibial SOS *z*-score was 0.31 (0.78) at baseline and 0.39 (0.79) at 1-year follow-up.

**Table 1:** Sample characteristics.

Variable	All participants (*n* = 109)
Sample characteristics at baseline	
Age, years	4.33 (0.61)
Girls, *n* (%)	48 (44.0)
Height, cm	107.35 (7.71)
Weight, kg	17.41 (3.02)
BMI, kg/m^2^	15.04 (1.50)
BMI-*z*	−0.27 (1.13)
BMI category, *n* (%)	
Underweight	3 (2.8)
Normal	100 (93.5)
Overweight and obese	4 (3.7)
SES, *n* (%)	
Low	37 (33.9)
Middle	36 (33.0)
High	36 (33.0)
24-h movement behavior	
SB, min/day	493.15 (75.80)
LPA, min/day	189.65 (39.17)
MVPA, min/day	46.62 (19.53)
Sleep, min/day	710.58 (90.93)
Total day duration, min/day	1431.01 (36.71)
Bone health	
Tibial SOS *z*-score, baseline	0.31 (0.78)
Tibial SOS *z*-score, 1-year follow-up	0.39 (0.79)

Data are mean (SD) or *n* (%). BMI, body mass index; BMI-*z*, WHO BMI-for-age *z*-score; MVPA, moderate-to-vigorous physical activity; LPA, light physical activity; SB, sedentary behavior; SES, socioeconomic status; SOS, speed of sound.

SB, LPA, MVPA, and sleep are the closed composition, rescaled so that the four parts sum to 1,440 min/day for every child; total day duration is the observed total before rescaling.

Weight was not recorded for two children, so BMI, BMI-*z* and the weight status category are available for 107 children and the weight status categories sum to 107.

### 3.2 Latent profile model selection

Latent profile models with one to five profiles were estimated and compared on the BIC, aBIC, entropy, the LMR, the BLRT, the size of the smallest profile, and the substantive interpretability of the resulting profiles (Table 2). Model fit improved markedly from one to three profiles: the BIC decreased to its minimum at the three-profile solution (BIC −22.950, aBIC −86.147), the addition of a third profile was supported by both the LMR (*p* = 0.020) and the BLRT (*p* = 0.004), and entropy rose from 0.747 to 0.894, indicating clearer separation between profiles. Extending the model to four profiles did not yield a comparable gain: the BIC increased (−12.423), entropy no longer improved (0.892), and, most importantly, the BLRT no longer supported the additional profile (*p* = 0.140), indicating that the four-profile model did not fit significantly better than the three-profile model. Although the aBIC continued to decrease and the LMR remained nominally significant for the fourth profile, greater weight was given to the BLRT and the BIC, which are regarded as more reliable indicators of the number of latent classes (Nylund et al., 2007). The smallest profile in the three-profile solution comprised 6.4% of the sample (*n* = 7); this exceeded the minimum size adopted in previous compositional latent profile work and was retained because it captured a behaviorally distinct long-sleeping subgroup of substantive interest (Balbim et al., 2024). Balancing statistical fit, parsimony and interpretability, the three-profile solution was selected.

**Table 2:** Fit indices of the latent 1–5-profile solutions.

Profiles	BIC	aBIC	Entropy	LMR *p*	BLRT *p*	Min class %
1	−1.049	−20.008	–	–	–	100.0
2	−18.353	−59.431	0.747	0.084	<0.001	38.5
3	−22.950	−86.147	0.894	0.020	0.004	6.4
4	−12.423	−97.740	0.892	0.015	0.140	6.4
5	12.306	−95.129	0.868	0.753	0.812	5.5

*N* = 109. BIC = Bayesian information criterion; aBIC = sample-size adjusted BIC; Entropy = classification certainty, with higher values indicating more precise assignment of children to profiles; LMR *p* = Lo–Mendell–Rubin likelihood ratio test *p*-value; BLRT *p* = bootstrapped likelihood ratio test *p*-value; Min class % reflects the minimum profile size in each solution.

### 3.3 Three-profile structure

The three profiles were distinguished by their SB, LPA, MVPA and sleep signatures (Figure 1). Relative to the sample mean, the Sedentary profile was characterised by above-average SB and below-average MVPA, the Low-active, long-sleeping profile by markedly above-average sleep together with below-average SB, LPA and MVPA, and the Active profile by above-average LPA and MVPA (Figure 1A). The average posterior probability of the assigned profile was 0.961, 0.988 and 0.950 for the Sedentary, the Low-active, long-sleeping and the Active profile, respectively (Supplementary Table S1).

**Figure 1:**
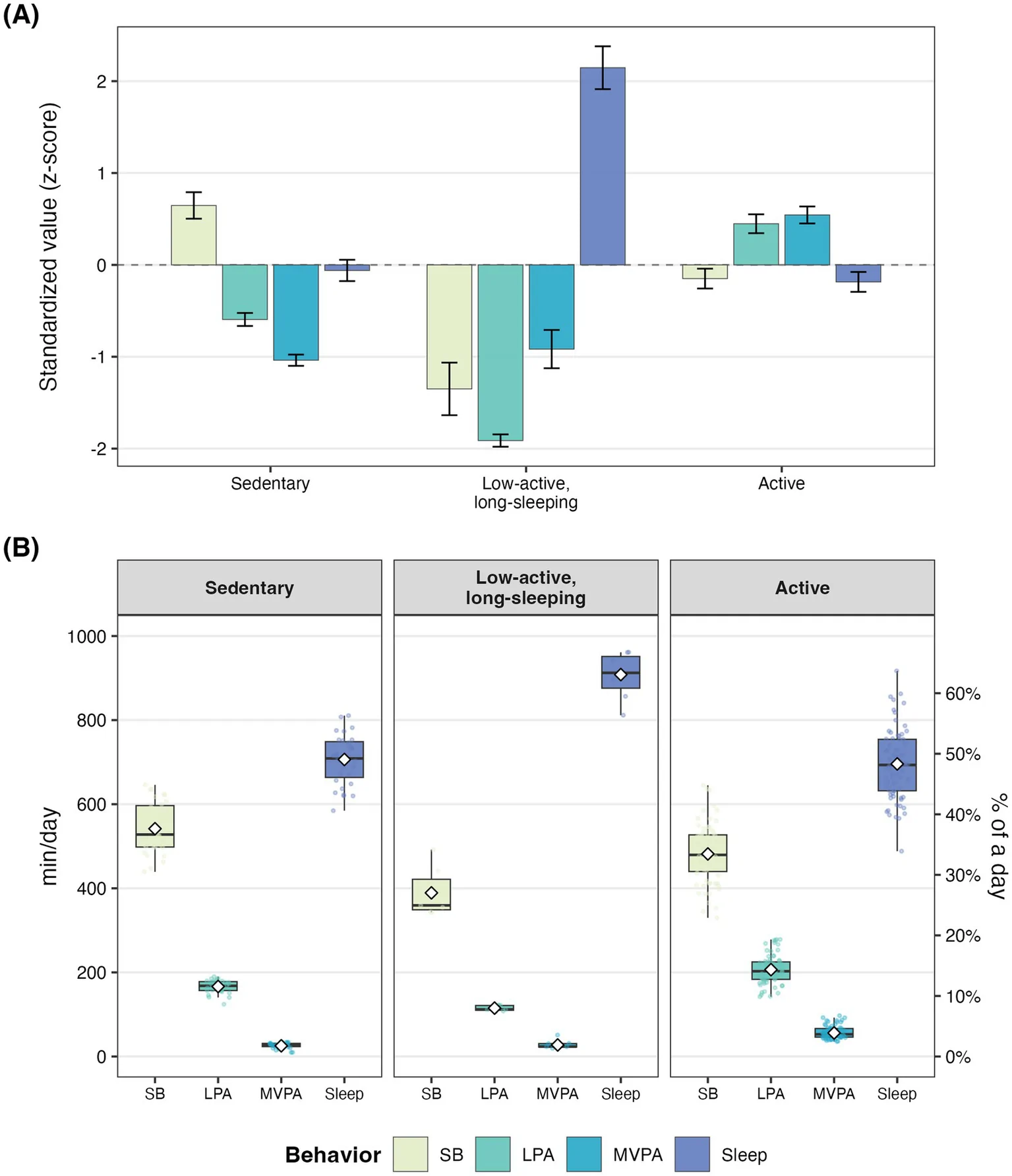
Behavioral signatures of the three 24-h movement behavior profiles. Profiles were derived from compositional latent profile analysis (*n* = 109). **(A)** Within-profile mean of each behavior expressed as a *z*-score standardised across the full sample (mean 0, SD 1); bars show the mean and error bars the standard error, and the dashed line marks the sample mean (*z* = 0). **(B)** Distribution of each behavior in minutes per day within each profile; boxes show the median and interquartile range, points individual children, and open diamonds the compositional mean of each behavior; the right axis expresses time as a percentage of the 24-h day. SB, sedentary behavior; LPA, light physical activity; MVPA, moderate-to-vigorous physical activity.

On the absolute scale (Figure 1B), the compositional mean of the largest profile, Active (*n* = 71, 65.1%), combined intermediate SB (481.71 min/day) with the highest LPA (206.53 min/day) and MVPA (55.98 min/day) and the shortest sleep (695.79 min/day). The Sedentary profile (*n* = 31, 28.4%) showed the highest SB (541.72 min/day), intermediate LPA (166.51 min/day), the lowest MVPA (25.40 min/day) and sleep of 706.36 min/day, which differed from the Active profile by 10.57 min/day. The small Low-active, long-sleeping profile (*n* = 7, 6.4%) combined the lowest SB (389.16 min/day) and LPA (115.08 min/day) with low MVPA (27.46 min/day) and markedly long sleep (908.29 min/day); all seven children in this profile slept longer than the 10–13 h/day recommended at this age (Tremblay et al., 2017).

### 3.4 Associations between covariates and profile membership

Associations between covariates and profile membership were examined using R3STEP, with the Sedentary profile as the reference (Figure 2). Relative to the Sedentary profile, the odds of belonging to the Active profile were higher for older children (OR 2.164 per year, 95% CI 1.012 to 4.630, *p* = 0.047) and did not differ by sex, SES score or BMI-*z* (*p* = 0.147, 0.378 and 0.267, respectively). Relative to the Sedentary profile, the odds of belonging to the Low-active, long-sleeping profile did not differ on any of the four covariates (*p* = 0.293 for age, 0.223 for sex, 0.175 for SES score and 0.102 for BMI-*z*).

**Figure 2:**
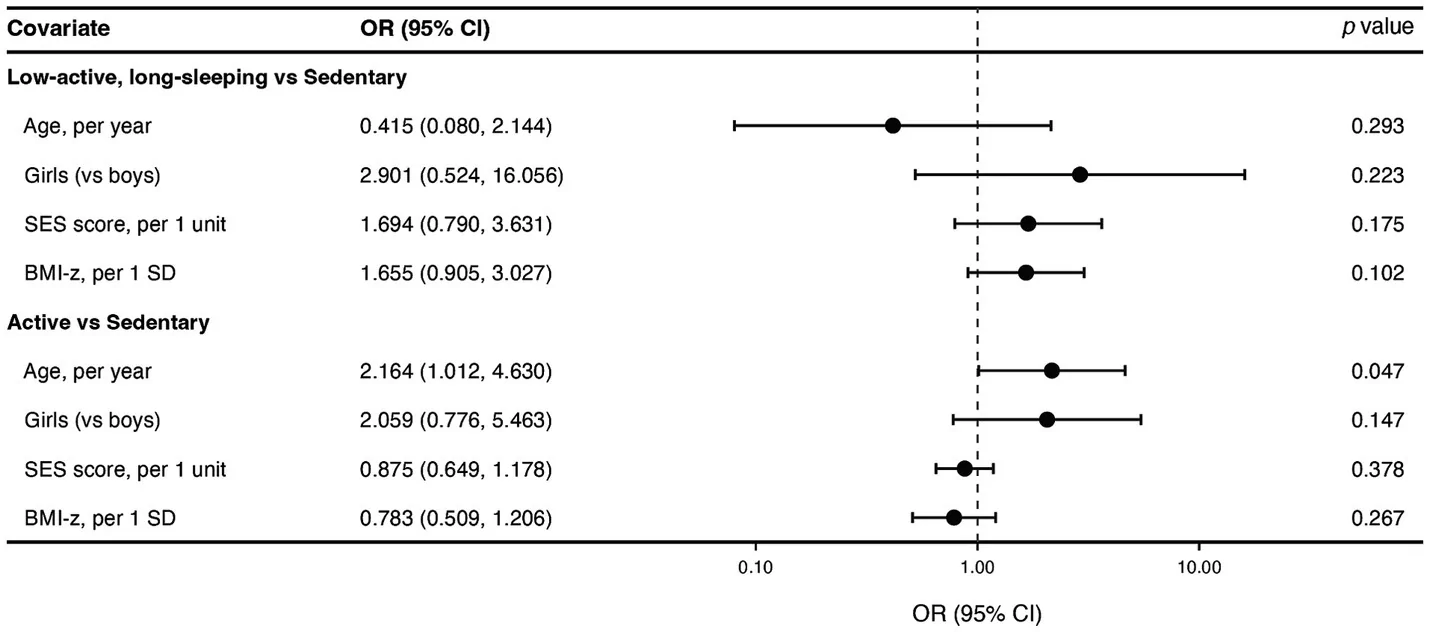
Associations between covariates and 24-h movement behavior profile membership. Forest plot of OR with 95% CI from the bias-adjusted three-step (R3STEP) approach, in which each covariate was entered separately to predict profile membership while accounting for classification uncertainty (*n* = 109 for age, sex and the SES score; *n* = 107 for BMI-*z*, which was not available for two children). The reference profile is Sedentary. ORs are expressed per year of age, for girls versus boys, per 1-unit increase in the SES score, and per 1 SD of BMI-*z*. OR, odds ratio; CI, confidence interval; BMI-*z*, WHO BMI-for-age *z*-score; SES, socioeconomic status.

### 3.5 Profile association with the tibial SOS *z*-score

As shown in Figure 3, in the model for the baseline score, adjusted for age, sex, SES score and BMI-*z*, neither profile differed from the Sedentary profile: the adjusted *β* was −0.323 (95% CI −1.033 to 0.387, *p* = 0.372) for the Low-active, long-sleeping profile and 0.137 (95% CI −0.237 to 0.511, *p* = 0.474) for the Active profile.

**Figure 3:**
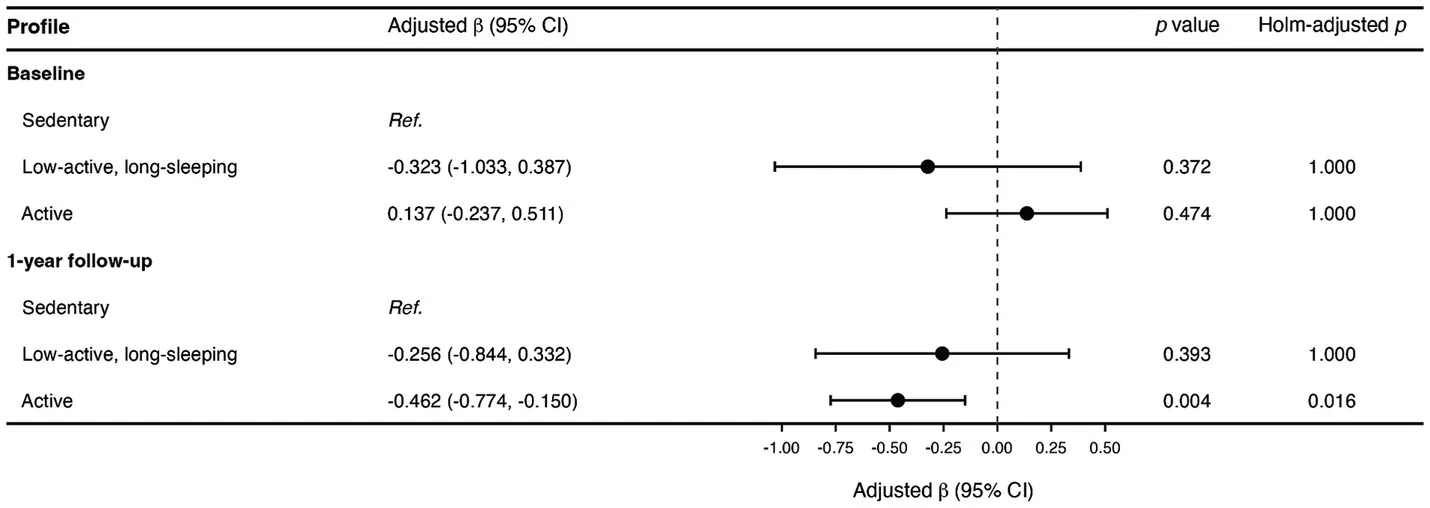
Adjusted associations between 24-h movement behavior profiles and the tibial SOS *z*-score at baseline and 1-year follow-up. Forest plot of adjusted difference (*β*) in the SOS *z*-score with 95% CI, relative to the Sedentary profile, estimated by the BCH approach (*n* = 109). Two children had no recorded weight and therefore no BMI-for-age *z*-score; they were retained by full information maximum likelihood. The model for the baseline score was adjusted for age, sex, SES score and BMI-*z*; the model for the 1-year follow-up score was adjusted for the same covariates and for the baseline score. CI, confidence interval. *p* values are shown before and after Holm adjustment over the four tests.

In the model for the follow-up score, adjusted for the baseline score as well as age, sex, SES score and BMI-*z*, children in the Active profile scored lower than children in the Sedentary profile (*β* − 0.462, 95% CI −0.774 to −0.150, *p* = 0.004). The Low-active, long-sleeping profile did not differ from the Sedentary profile (*β* −0.256, 95% CI −0.844 to 0.332, *p* = 0.393). After Holm adjustment over the four tests, the contrast for the Active profile remained significant (*p* = 0.016). The smallest difference detectable at 80% power was 0.409 SOS *z*-score units for the Active contrast and 0.794 for the Low-active, long-sleeping contrast, against observed differences of −0.462 and −0.256 (Supplementary Table S2).

### 3.6 Sensitivity analyses

The profile structure was re-estimated with two and with four profiles, using the same preprocessing and the same estimation settings as the primary analysis (Supplementary Table S3). In the two-profile solution the 31 children of the Sedentary profile, six of the seven children of the Low-active, long-sleeping profile and five children of the Active profile formed one class of 42, and the remaining 67 children formed the other. In the four-profile solution the Sedentary profile (*n* = 31) and the Low-active, long-sleeping profile (*n* = 7) were each reproduced as a separate class, and the Active profile divided into a class of 49 with an MVPA compositional mean of 48.68 min/day and a class of 22 with 76.08 min/day. Within the Low-active, long-sleeping profile the posterior probability of the assigned profile was at least 0.934 for every child (Supplementary Table S1).

The association with the tibial SOS *z*-score was re-estimated after assigning each child to the profile with the highest posterior probability, using HC3 standard errors (Supplementary Table S4). At baseline the adjusted differences were −0.331 (*p* = 0.440) for the Low-active, long-sleeping profile and 0.141 (*p* = 0.448) for the Active profile. At the 1-year follow-up they were −0.232 (*p* = 0.508) and −0.383 (95% CI −0.695 to −0.071, *p* = 0.017); after Holm adjustment over the four tests of this specification the latter was 0.067. Excluding the Low-active, long-sleeping profile gave −0.392 (95% CI −0.702 to −0.081, *p* = 0.014, *n* = 100). Under the two- and four-profile solutions the same contrast was estimated by the same BCH procedure (Supplementary Table S5). In the two-profile solution the class of 67 differed from the class of 42 by −0.466 (95% CI −0.793 to −0.139, *p* = 0.005). In the four-profile solution the two classes into which the Active profile divided differed from the class of 31 by −0.424 (*p* = 0.012) and −0.475 (*p* = 0.014), and the class of seven differed by −0.242 (*p* = 0.408).

## 4 Discussion

This prospective analysis of preschool children identified three profiles of the 24-h movement composition, each characterised by its SB, LPA, MVPA and sleep signature: a Sedentary profile, a Low-active, long-sleeping profile and an Active profile. At baseline the tibial SOS *z*-score did not differ across the three profiles. At the 1-year follow-up, after adjustment for the baseline score, age, sex, SES and BMI-*z*, children in the Active profile scored 0.462 SOS *z*-score units lower than children in the Sedentary profile (95% CI −0.774 to −0.150; Holm-adjusted *p* = 0.016). The Low-active, long-sleeping profile did not differ from the Sedentary profile (−0.256, 95% CI −0.844 to 0.332); with seven children, the smallest difference detectable in that contrast was 0.794 units, about three times the difference observed. Older children were more likely to belong to the Active profile than to the Sedentary profile, whereas sex, SES and BMI-*z* were not associated with profile membership.

The contribution of this analysis is to characterise differences in daily time allocation that guideline adherence categories do not explicitly describe. The earlier study classified children by whether they met recommendations for physical activity, screen time and sleep (Li et al., 2024). Here, profiles incorporated accelerometer-derived SB, distinguished LPA from MVPA, and expressed all four behaviors as proportions of the fixed 24-h day. For example, the Sedentary and Low-active, long-sleeping profiles had similarly low MVPA but differed markedly in their allocation of time to SB and sleep. Relating these combinations to the follow-up SOS *z*-score provides a complementary description of behavioral variation associated with bone health.

The most notable finding was that the profile with the greatest amount of activity had the lower tibial SOS *z*-score 1 year later. Children in the Active profile accumulated the most MVPA (55.98 min/day) and the most LPA (206.53 min/day) of the three profiles, yet scored 0.462 SOS *z*-score units below the Sedentary profile at follow-up. This appears to run counter to the prevailing evidence that higher amounts of physical activity are associated with better indicators of bone health in children aged 3–6 years (Pate et al., 2019), and to the earlier analysis of these children, in which meeting the physical activity guideline was associated with a lower risk of insufficient bone status 1 year later (Li et al., 2024). Within a compositional framework, however, time spent in one behavior is necessarily taken from another, so that this profile does not represent more activity with all else held constant. Relative to the Sedentary profile, the approximately 71 min/day of additional LPA and MVPA in the Active profile came from approximately 60 min/day less SB and 11 min/day less sleep, and this profile had the shortest sleep of the three (695.79 min/day). Sleep duration and physical activity have been shown to interact in their association with bone strength in preschoolers, such that the benefit of activity is conditional on sleep being adequate (Fu et al., 2023). The difference observed here may therefore reflect the allocation of the whole day rather than the amount of activity alone. Consistent with this, compositional analyses have found the differences in bone mineral density attributable to MVPA to be small (Taylor et al., 2018), and exercise added beyond the activity children already undertake has not been shown to improve densitometric measures of bone health (McCaskie et al., 2023). An alternative explanation concerns the outcome measure: bone status was quantified as tibial SOS, and physical activity has been reported to relate to broadband ultrasound attenuation and the stiffness index without a detectable influence on SOS (De Smet et al., 2015), while an earlier study observed lower ultrasound transmission velocity in more active prepubertal girls (Lappe et al., 1998).

The three profiles also differed in sleep in a way that placed one at the centre and two at the extremes of the observed range, with sleep of 706.36 min/day in the Sedentary profile, 908.29 min/day in the Low-active, long-sleeping profile and 695.79 min/day in the Active profile. A contribution of sleep to skeletal status is biologically plausible: 3 weeks of sleep restriction with concurrent circadian disruption lowered the bone formation marker P1NP without a corresponding change in resorption, indicating uncoupling of bone turnover, and the decrease was greater in younger than in older men (Swanson et al., 2017); in children, longer sleep duration and daytime napping have been associated with higher bone mineral content (Casazza et al., 2011). The contrast at the short-sleeping end of the present sample was nevertheless small, since the Active profile slept only 10.57 min/day less than the Sedentary profile and both durations fall within the range recommended for this age. Sleep duration alone is therefore unlikely to account for the difference at that end, which is more consistent with the whole-day compositional reading advanced above. At the long-sleeping end, the profile combined markedly long sleep with the lowest SB (389.16 min/day) and LPA (115.08 min/day) and low MVPA (27.46 min/day), leaving the least waking time available for mechanical loading; quantitative ultrasound measures have been reported to differ across levels of physical activity in preschool children (Madic et al., 2017), but the follow-up score of this profile was not distinguishable from that of the Sedentary profile, so no inference about long sleep can be drawn from it. Both non-reference profiles scored lower than the Sedentary profile at follow-up, but only the contrast for the Active profile was distinguishable from zero, so a more balanced allocation of the day being more favourable for bone than allocations extreme in either direction remains an interpretation rather than a tested result.

Of the four covariates examined, only age was associated with profile membership. Older children were more likely to belong to the Active profile, which is consistent with the transition out of daytime napping, described as a universal process in human development and a normal part of maturation in early childhood (Spencer and Riggins, 2022); as naps are relinquished, total sleep shortens and more of the day becomes available for activity. This direction differs from that reported in an older cohort followed from 5.5 to 8 years, in which boys were more likely to belong to a highly active profile and most children moved toward less active, longer-sleeping profiles with age (Padmapriya et al., 2021), which suggests that the association between age and profile membership may be specific to the period during which napping is being relinquished. Sex, SES and BMI-*z* were not associated with profile membership. Differences in daily physical activity and sedentary time between children who are and are not overweight (Sigmund et al., 2024), and sociodemographic differences in meeting movement guidelines (Kracht et al., 2019), have been documented in other samples, so the absence of such differences here may reflect the narrow socioeconomic range of the participating kindergartens and the limited sample size rather than the absence of any association.

This study has several strengths. The 24-h day was treated as a closed composition and summarised through person-centred profiles, so that children were grouped by the pattern of the whole day rather than by their attainment of separate behavioral thresholds. Behavior and bone were assessed prospectively, with bone measured both at baseline and 1 year later, which allowed the absence of a difference at baseline and the divergence at follow-up to be observed within the same children. SB and physical activity were device-measured over seven consecutive days using hip-worn accelerometry fitted by trained researchers, sleep was reported daily by parents and collated by class teachers and corroborated against the accelerometer wear log rather than relying on a single retrospective report, and classification uncertainty was carried into both the covariate models and the outcome models. Several limitations qualify these findings. The sample was small, and the Low-active, long-sleeping profile comprised only seven children, so that its estimate was imprecise and should be regarded as descriptive rather than inferential. These seven children were separated clearly within the present sample, each with a posterior probability of at least 0.934 for the assigned profile, and they again formed a separate class when four profiles were fitted. The design was observational, so that causal inference is not warranted and residual confounding by unmeasured factors such as dietary calcium and vitamin D intake or sun exposure cannot be excluded. The mean number of valid days, the mean daily wear time and the non-wear time were not reported in this present analysis, so the extent to which non-wear time was absorbed into the closed composition could not be quantified. Bone health was quantified as tibial SOS by quantitative ultrasound, which differs from dual-energy X-ray absorptiometry and peripheral quantitative computed tomography in the properties it captures (Binkley et al., 2008). Future studies would therefore benefit from larger and preferably multi-centre samples in which small but behaviorally distinct profiles can be estimated with greater precision, from longer follow-up in which changes in profile membership are modelled rather than assumed, and from designs that pair ultrasound with densitometric measures and record dietary and sun-exposure information.

## 5 Conclusion

Among preschool children, three profiles of the 24-h movement composition were identified. The profiles were unrelated to the tibial SOS *z*-score at baseline, but 1 year later children in the Active profile scored lower than children in the Sedentary profile. Attention to the 24-h movement composition as a whole may therefore be important when bone health is promoted in early childhood.

## Data Availability

The raw data supporting the conclusions of this article will be made available by the authors, without undue reservation.
