# Supplementary material for: Latent profiles of 24-h movement behaviors and bone health at one-year follow-up in Chinese preschool children
Source: Front Psychol. 2026 Oct 13;17:1964754. doi: 10.3389/fpsyg.2026.1964754 (PMC13710083; doi:10.3389/fpsyg.2026.1964754)
Supplement: Supplementary file 1 [file Data_Sheet_1.PDF]

SUPPLEMENTARY MATERIAL

Table of Contents

Table S1. Posterior classification probabilities for the primary three-profile solution

Table S2. Minimum detectable difference in the tibial SOS z-score for each profile contrast

Table S3. Profile characteristics and correspondence across the two-, three- and four-profile solutions

Table S4. Sensitivity analyses based on modal profile assignment

Table S5. Associations of profile membership with follow-up tibial SOS z-score under the two- and four-profile solutions

Table S1. Posterior classification probabilities for the primary three-profile solution

Most likely profile	n	Sedentary profile, mean	Low-active, long-sleeping profile, mean	Active profile, mean	Assigned-profile probability		Probability	
					median	min–max	≥ 0.70, n (%)	≥ 0.80, n (%)
Sedentary	31	0.961	0.000	0.039	0.994	0.825–1.000	31 (100.0)	31 (100.0)
Low-active, long-sleeping	7	0.001	0.988	0.012	0.996	0.934–1.000	7 (100.0)	7 (100.0)
Active	71	0.050	0.000	0.950	0.999	0.552–1.000	66 (93.0)	65 (91.5)

Note. N = 109. Rows denote modal profile assignment based on the highest posterior probability. The first three probability columns form the average posterior-probability matrix, with diagonal values indicating the mean probability of membership in the assigned profile.

Table S2. Minimum detectable difference in the tibial SOS z-score for each profile contrast

Contrast	Group sizes	Observed difference	Min detectable difference
Active versus Sedentary	71 vs 31	-0.462	0.409
Low-active, long-sleeping versus Sedentary	7 vs 31	-0.256	0.794

Note. Values are expressed in SOS z-score units, corresponding to standard deviations of the age-, sex- and ethnicity-specific reference population. Minimum detectable differences were calculated using the observed analytic group sizes and a residual standard deviation of 0.678 from the follow-up modal-class model, assuming 80% power and an unadjusted two-sided α of 0.05. Observed differences are the baseline-adjusted BCH estimates at the 1-year follow-up.

Table S3. Profile characteristics and correspondence across the two-, three- and four-profile solutions

Profile	n (%)	SB, min/day	LPA, min/day	MVPA, min/day	Sleep, min/day	From Sedentary profile, n	From Low-active, long-sleeping profile, n	From Active profile, n
Two-profile solution								
Class 1	67 (61.5)	482.28	208.63	57.44	691.66	0	1	66
Class 2	42 (38.5)	508.68	158.09	26.77	746.47	31	6	5
Three-profile solution								
Active	71 (65.1)	481.71	206.53	55.98	695.79	0	0	71
Sedentary	31 (28.4)	541.72	166.51	25.40	706.36	31	0	0
Low-active, long-sleeping	7 (6.4)	389.16	115.08	27.46	908.29	0	7	0
Four-profile solution								
Class 1	49 (45.0)	485.41	199.03	48.68	706.87	0	0	49
Class 2	31 (28.4)	541.72	166.51	25.40	706.36	31	0	0
Class 3	22 (20.2)	471.62	223.34	76.08	668.96	0	0	22
Class 4	7 (6.4)	389.16	115.08	27.46	908.29	0	7	0

Note. N = 109. Values for SB, LPA, MVPA and sleep are compositional means, calculated by closing the vector of componentwise geometric means to 1,440 min/day. Classes in the two- and four-profile solutions are numbered in descending order of size and have no substantive labels. The correspondence columns show the numbers of children from each primary three-profile group assigned to each class. SB = sedentary behaviour; LPA = light physical activity; MVPA = moderate-to-vigorous physical activity.

Table S4. Sensitivity analyses based on modal profile assignment

Analysis specification	Contrast	Analysis sample, n	Adjusted mean difference	HC3 standard error	95% CI	Unadjusted p value	Holm-adjusted p value
Modal profile assignment	**Baseline**						
	Low-active, long-sleeping vs Sedentary	107	-0.331	0.427	-1.179, 0.517	0.440	1.000
	Active vs Sedentary	107	0.141	0.185	-0.226, 0.507	0.448	1.000
	**1-year follow-up**						
	Low-active, long-sleeping vs Sedentary	107	-0.232	0.349	-0.925, 0.461	0.508	1.000
	Active vs Sedentary	107	-0.383	0.157	-0.695, -0.071	0.017	0.067
Excluding the smallest profile	**1-year follow-up**						
	Active vs Sedentary	100	-0.392	0.156	-0.702, -0.081	0.014	Not applicable

Note. Children were assigned to their most likely profile based on the highest posterior probability, and linear models were fitted using HC3 heteroskedasticity-robust standard errors. Baseline models were adjusted for age, sex, SES score and BMI-for-age z-score; follow-up models were additionally adjusted for baseline SOS z-score. Estimates are adjusted mean differences in SOS z-score relative to the Sedentary profile. The four full-sample contrasts were adjusted using the Holm procedure, whereas the analysis excluding the smallest profile contained one prespecified contrast and was not adjusted.

Weight, and therefore the BMI-for-age z-score, was not recorded for two children; as these models were fitted in R with listwise deletion, they include 107 of the 109 children, and 100 of the 102 remaining after the smallest profile was excluded.

Table S5. Associations of profile membership with follow-up tibial SOS z-score under the two- and four-profile solutions

Target profile	Reference profile	Adjusted mean difference	Standard error	95% CI	p value	Overall Wald chi-square	df	Overall p value
Two-profile solution								
Class 1 (n = 67)	Class 2 (n = 42)	-0.466	0.167	-0.793, -0.139	0.005	7.783	1	0.005
Four-profile solution								
Class 1 (n = 49)	Class 2 (n = 31)	-0.424	0.168	-0.753, -0.095	0.012	8.748	3	0.033
Class 3 (n = 22)	Class 2 (n = 31)	-0.475	0.194	-0.855, -0.095	0.014	-	-	-
Class 4 (n = 7)	Class 2 (n = 31)	-0.242	0.292	-0.814, 0.330	0.408	-	-	-

Note. N = 109. The two children without a BMI-for-age z-score were retained by full information maximum likelihood.

Estimates are adjusted mean differences in SOS z-score obtained using the BCH method, controlling for baseline SOS z-score, age, sex, the continuous socioeconomic status PC1 score and BMI-for-age z-score. Classes are numbered in descending order of size. Within each solution, the class with the lowest compositional mean for MVPA was used as the reference, consistent with the rule used to identify the Sedentary profile in the primary solution. The overall Wald test evaluates the joint null hypothesis that the adjusted means are equal across classes.

Mplus input files

Latent profile analysis, one profile

```
TITLE: LPA K1;
DATA: FILE = mp.dat;
VARIABLE: NAMES = id ilr1 ilr2 ilr3 sosz1 sosz2 age sexf ses bmiz;
  USEVARIABLES = ilr1 ilr2 ilr3;
  IDVARIABLE = id;
  CLASSES = c(1);
  MISSING = ALL (-999);
ANALYSIS: TYPE = MIXTURE; ESTIMATOR = MLR;
  STARTS = 500 50; MITERATIONS = 5000;
MODEL:
  %OVERALL%
  %c#1%
  [ilr1-ilr3]; ilr1-ilr3;
OUTPUT: ;
```

Latent profile analysis, two profiles

```
TITLE: LPA K2;
DATA: FILE = mp.dat;
VARIABLE: NAMES = id ilr1 ilr2 ilr3 sosz1 sosz2 age sexf ses bmiz;
  USEVARIABLES = ilr1 ilr2 ilr3;
  IDVARIABLE = id;
  CLASSES = c(2);
  MISSING = ALL (-999);
ANALYSIS: TYPE = MIXTURE; ESTIMATOR = MLR;
  STARTS = 500 50; MITERATIONS = 5000;
  LRTBOOTSTRAP = 500;
MODEL:
  %OVERALL%
  %c#1%
  [ilr1-ilr3]; ilr1-ilr3;
  %c#2%
  [ilr1-ilr3]; ilr1-ilr3;
OUTPUT: TECH11 TECH14;
```

Latent profile analysis, three profiles

```
TITLE: LPA K3;
DATA: FILE = mp.dat;
VARIABLE: NAMES = id ilr1 ilr2 ilr3 sosz1 sosz2 age sexf ses bmiz;
  USEVARIABLES = ilr1 ilr2 ilr3;
  IDVARIABLE = id;
  CLASSES = c(3);
  MISSING = ALL (-999);
ANALYSIS: TYPE = MIXTURE; ESTIMATOR = MLR;
  STARTS = 500 50; MITERATIONS = 5000;
  LRTBOOTSTRAP = 500;
MODEL:
  %OVERALL%
  %c#1%
  [ilr1-ilr3]; ilr1-ilr3;
  %c#2%
  [ilr1-ilr3]; ilr1-ilr3;
  %c#3%
  [ilr1-ilr3]; ilr1-ilr3;
OUTPUT: TECH11 TECH14;
```

Latent profile analysis, four profiles

```
TITLE: LPA K4;
DATA: FILE = mp.dat;
VARIABLE: NAMES = id ilr1 ilr2 ilr3 sosz1 sosz2 age sexf ses bmiz;
```

```
  USEVARIABLES = ilr1 ilr2 ilr3;
  IDVARIABLE = id;
  CLASSES = c(4);
  MISSING = ALL (-999);
ANALYSIS: TYPE = MIXTURE; ESTIMATOR = MLR;
  STARTS = 500 50; MITERATIONS = 5000;
  LRTBOOTSTRAP = 500;
MODEL:
  %OVERALL%
  %c#1%
  [ilr1-ilr3]; ilr1-ilr3;
  %c#2%
  [ilr1-ilr3]; ilr1-ilr3;
  %c#3%
  [ilr1-ilr3]; ilr1-ilr3;
  %c#4%
  [ilr1-ilr3]; ilr1-ilr3;
OUTPUT: TECH11 TECH14;
```

Latent profile analysis, five profiles

```
TITLE: LPA K5;
DATA: FILE = mp.dat;
VARIABLE: NAMES = id ilr1 ilr2 ilr3 sosz1 sosz2 age sexf ses bmiz;
  USEVARIABLES = ilr1 ilr2 ilr3;
  IDVARIABLE = id;
  CLASSES = c(5);
  MISSING = ALL (-999);
ANALYSIS: TYPE = MIXTURE; ESTIMATOR = MLR;
  STARTS = 500 50; MITERATIONS = 5000;
  LRTBOOTSTRAP = 500;
MODEL:
  %OVERALL%
  %c#1%
  [ilr1-ilr3]; ilr1-ilr3;
  %c#2%
  [ilr1-ilr3]; ilr1-ilr3;
  %c#3%
  [ilr1-ilr3]; ilr1-ilr3;
  %c#4%
  [ilr1-ilr3]; ilr1-ilr3;
  %c#5%
  [ilr1-ilr3]; ilr1-ilr3;
OUTPUT: TECH11 TECH14;
```

Three-profile solution, posterior probabilities saved

```
TITLE: LPA K3 final;
DATA: FILE = mp.dat;
VARIABLE: NAMES = id ilr1 ilr2 ilr3 sosz1 sosz2 age sexf ses bmiz;
  USEVARIABLES = ilr1 ilr2 ilr3;
  IDVARIABLE = id;
  CLASSES = c(3);
  MISSING = ALL (-999);
ANALYSIS: TYPE = MIXTURE; ESTIMATOR = MLR; STARTS = 500 50; MITERATIONS =
5000;
MODEL:
  %OVERALL%
  %c#1%
  [ilr1-ilr3]; ilr1-ilr3;
  %c#2%
  [ilr1-ilr3]; ilr1-ilr3;
  %c#3%
  [ilr1-ilr3]; ilr1-ilr3;
OUTPUT: ;
SAVEDATA: FILE = cprob.dat; SAVE = CPROBABILITIES;
```

R3STEP, age

```
TITLE: R3STEP age;
DATA: FILE = mp.dat;
VARIABLE: NAMES = id ilr1 ilr2 ilr3 sosz1 sosz2 age sexf ses bmiz;
  USEVARIABLES = ilr1 ilr2 ilr3;
  IDVARIABLE = id;
  CLASSES = c(3);
  AUXILIARY = age (R3STEP); MISSING = ALL (-999);
ANALYSIS: TYPE = MIXTURE; ESTIMATOR = MLR; STARTS = 500 50; MITERATIONS =
5000;
MODEL:
  %OVERALL%
  %c#1%
  [ilr1-ilr3]; ilr1-ilr3;
  %c#2%
  [ilr1-ilr3]; ilr1-ilr3;
  %c#3%
  [ilr1-ilr3]; ilr1-ilr3;
OUTPUT: ;
```

R3STEP, sex

```
TITLE: R3STEP sexf;
DATA: FILE = mp.dat;
VARIABLE: NAMES = id ilr1 ilr2 ilr3 sosz1 sosz2 age sexf ses bmiz;
  USEVARIABLES = ilr1 ilr2 ilr3;
  IDVARIABLE = id;
  CLASSES = c(3);
  AUXILIARY = sexf (R3STEP); MISSING = ALL (-999);
ANALYSIS: TYPE = MIXTURE; ESTIMATOR = MLR; STARTS = 500 50; MITERATIONS =
5000;
MODEL:
  %OVERALL%
  %c#1%
  [ilr1-ilr3]; ilr1-ilr3;
  %c#2%
  [ilr1-ilr3]; ilr1-ilr3;
  %c#3%
  [ilr1-ilr3]; ilr1-ilr3;
OUTPUT: ;
```

R3STEP, socioeconomic status score

```
TITLE: R3STEP ses;
DATA: FILE = mp.dat;
VARIABLE: NAMES = id ilr1 ilr2 ilr3 sosz1 sosz2 age sexf ses bmiz;
  USEVARIABLES = ilr1 ilr2 ilr3;
  IDVARIABLE = id;
  CLASSES = c(3);
  AUXILIARY = ses (R3STEP); MISSING = ALL (-999);
ANALYSIS: TYPE = MIXTURE; ESTIMATOR = MLR; STARTS = 500 50; MITERATIONS =
5000;
MODEL:
  %OVERALL%
  %c#1%
  [ilr1-ilr3]; ilr1-ilr3;
  %c#2%
  [ilr1-ilr3]; ilr1-ilr3;
  %c#3%
  [ilr1-ilr3]; ilr1-ilr3;
OUTPUT: ;
```

R3STEP, BMI-for-age z-score

```
TITLE: R3STEP bmiz;
DATA: FILE = mp.dat;
VARIABLE: NAMES = id ilr1 ilr2 ilr3 sosz1 sosz2 age sexf ses bmiz;
  USEVARIABLES = ilr1 ilr2 ilr3;
  IDVARIABLE = id;
  CLASSES = c(3);
```

```
  AUXILIARY = bmiz (R3STEP); MISSING = ALL (-999);
ANALYSIS: TYPE = MIXTURE; ESTIMATOR = MLR; STARTS = 500 50; MITERATIONS =
5000;
MODEL:
  %OVERALL%
  %c#1%
  [ilr1-ilr3]; ilr1-ilr3;
  %c#2%
  [ilr1-ilr3]; ilr1-ilr3;
  %c#3%
  [ilr1-ilr3]; ilr1-ilr3;
OUTPUT: ;
```

BCH step 1, weights saved

```
TITLE: LPA K3 final;
DATA: FILE = mp.dat;
VARIABLE: NAMES = id ilr1 ilr2 ilr3 sosz1 sosz2 age sexf ses bmiz;
  USEVARIABLES = ilr1 ilr2 ilr3;
  IDVARIABLE = id;
  CLASSES = c(3);
  MISSING = ALL (-999);
ANALYSIS: TYPE = MIXTURE; ESTIMATOR = MLR; STARTS = 500 50; MITERATIONS =
5000;
MODEL:
  %OVERALL%
  %c#1%
  [ilr1-ilr3]; ilr1-ilr3;
  %c#2%
  [ilr1-ilr3]; ilr1-ilr3;
  %c#3%
  [ilr1-ilr3]; ilr1-ilr3;
OUTPUT: ;
SAVEDATA: FILE = bchw.dat; SAVE = BCHWEIGHTS;
```

BCH step 2, baseline tibial SOS z-score

```
TITLE: BCH baseline covariate adjusted;
DATA: FILE = mp2.dat;
VARIABLE: NAMES = id sosz2 sosz1 age sexf ses bmiz w1 w2 w3;
  USEVARIABLES = sosz1 age sexf ses bmiz w1 w2 w3;
  IDVARIABLE = id;
  CLASSES = c(3);
  TRAINING = w1 w2 w3 (BCH); MISSING = ALL (-999);
ANALYSIS: TYPE = MIXTURE; ESTIMATOR = MLR; STARTS = 0;
MODEL:
  %OVERALL%
  sosz1 ON age sexf ses bmiz;
  age sexf ses bmiz;
  %c#1%
  [sosz1] (m1);
  sosz1;
  %c#2%
  [sosz1] (m2);
  sosz1;
  %c#3%
  [sosz1] (m3);
  sosz1;
MODEL CONSTRAINT:
  NEW(dlong dactive);
  dlong   = m2 - m1;
  dactive = m3 - m1;
OUTPUT: ;
```

BCH step 2, follow-up tibial SOS z-score

```
TITLE: BCH covariate and baseline adjusted;
DATA: FILE = mp2.dat;
VARIABLE: NAMES = id sosz2 sosz1 age sexf ses bmiz w1 w2 w3;
```

```
  USEVARIABLES = sosz2 sosz1 age sexf ses bmiz w1 w2 w3;
  IDVARIABLE = id;
  CLASSES = c(3);
  TRAINING = w1 w2 w3 (BCH); MISSING = ALL (-999);
ANALYSIS: TYPE = MIXTURE; ESTIMATOR = MLR; STARTS = 0;
MODEL:
  %OVERALL%
  sosz2 ON sosz1 age sexf ses bmiz;
  sosz1 age sexf ses bmiz;
  %c#1%
  [sosz2] (m1);
  sosz2;
  %c#2%
  [sosz2] (m2);
  sosz2;
  %c#3%
  [sosz2] (m3);
  sosz2;
MODEL CONSTRAINT:
  NEW(dlong dactive);
  dlong   = m2 - m1;
  dactive = m3 - m1;
OUTPUT: ;
```

Sensitivity analysis, two-profile solution

```
TITLE: mainK2;
DATA: FILE = mainK2.dat;
VARIABLE: NAMES = id ilr1 ilr2 ilr3;
  USEVARIABLES = ilr1 ilr2 ilr3;
  IDVARIABLE = id;
  CLASSES = c(2);
  MISSING = ALL (-999);
ANALYSIS: TYPE = MIXTURE; ESTIMATOR = MLR;
  STARTS = 500 50; MITERATIONS = 5000;
  LRTBOOTSTRAP = 500;
MODEL:
  %OVERALL%
  %c#1%
  [ilr1-ilr3]; ilr1-ilr3;
  %c#2%
  [ilr1-ilr3]; ilr1-ilr3;
OUTPUT: TECH11 TECH14;
SAVEDATA: FILE = mainK2_out.dat; SAVE = CPROBABILITIES;
```

Sensitivity analysis, four-profile solution

```
TITLE: mainK4;
DATA: FILE = mainK4.dat;
VARIABLE: NAMES = id ilr1 ilr2 ilr3;
  USEVARIABLES = ilr1 ilr2 ilr3;
  IDVARIABLE = id;
  CLASSES = c(4);
  MISSING = ALL (-999);
ANALYSIS: TYPE = MIXTURE; ESTIMATOR = MLR;
  STARTS = 500 50; MITERATIONS = 5000;
  LRTBOOTSTRAP = 500;
MODEL:
  %OVERALL%
  %c#1%
  [ilr1-ilr3]; ilr1-ilr3;
  %c#2%
  [ilr1-ilr3]; ilr1-ilr3;
  %c#3%
  [ilr1-ilr3]; ilr1-ilr3;
  %c#4%
  [ilr1-ilr3]; ilr1-ilr3;
OUTPUT: TECH11 TECH14;
```

```
SAVEDATA: FILE = mainK4_out.dat; SAVE = CPROBABILITIES;
```

Sensitivity analysis, BCH weights, two-profile solution

```
TITLE: bchK2_w;
DATA: FILE = bchK2_w.dat;
VARIABLE: NAMES = id ilr1 ilr2 ilr3;
  USEVARIABLES = ilr1 ilr2 ilr3;
  IDVARIABLE = id;
  CLASSES = c(2);
  MISSING = ALL (-999);
ANALYSIS: TYPE = MIXTURE; ESTIMATOR = MLR;
  STARTS = 500 50; MITERATIONS = 5000;
  LRTBOOTSTRAP = 500;
MODEL:
  %OVERALL%
  %c#1%
  [ilr1-ilr3]; ilr1-ilr3;
  %c#2%
  [ilr1-ilr3]; ilr1-ilr3;
OUTPUT: TECH11 TECH14;
SAVEDATA: FILE = bchK2_w_out.dat; SAVE = BCHWEIGHTS;
```

Sensitivity analysis, BCH weights, four-profile solution

```
TITLE: bchK4_w;
DATA: FILE = bchK4_w.dat;
VARIABLE: NAMES = id ilr1 ilr2 ilr3;
  USEVARIABLES = ilr1 ilr2 ilr3;
  IDVARIABLE = id;
  CLASSES = c(4);
  MISSING = ALL (-999);
ANALYSIS: TYPE = MIXTURE; ESTIMATOR = MLR;
  STARTS = 500 50; MITERATIONS = 5000;
  LRTBOOTSTRAP = 500;
MODEL:
  %OVERALL%
  %c#1%
  [ilr1-ilr3]; ilr1-ilr3;
  %c#2%
  [ilr1-ilr3]; ilr1-ilr3;
  %c#3%
  [ilr1-ilr3]; ilr1-ilr3;
  %c#4%
  [ilr1-ilr3]; ilr1-ilr3;
OUTPUT: TECH11 TECH14;
SAVEDATA: FILE = bchK4_w_out.dat; SAVE = BCHWEIGHTS;
```

Sensitivity analysis, BCH outcome model, two-profile solution

```
TITLE: BCH K2 follow-up;
DATA: FILE = bchK2.dat;
VARIABLE: NAMES = id sosz2 sosz1 age sexf ses bmiz w1 w2;
  USEVARIABLES = sosz2 sosz1 age sexf ses bmiz w1 w2;
  IDVARIABLE = id;
  CLASSES = c(2);
  TRAINING = w1 w2 (BCH); MISSING = ALL (-999);
ANALYSIS: TYPE = MIXTURE; ESTIMATOR = MLR; STARTS = 0;
MODEL:
  %OVERALL%
  sosz2 ON sosz1 age sexf ses bmiz;
  sosz1 age sexf ses bmiz;
  %c#1%
  [sosz2] (m1);
  sosz2;
  %c#2%
  [sosz2] (m2);
  sosz2;
MODEL CONSTRAINT:
```

```
NEW(d2);
d2 = m2 - m1;
MODEL TEST:
  0 = m2 - m1;
OUTPUT: ;
```

Sensitivity analysis, BCH outcome model, four-profile solution

```
TITLE: BCH K4 follow-up;
DATA: FILE = bchK4.dat;
VARIABLE: NAMES = id sosz2 sosz1 age sexf ses bmiz w1 w2 w3 w4;
  USEVARIABLES = sosz2 sosz1 age sexf ses bmiz w1 w2 w3 w4;
  IDVARIABLE = id;
  CLASSES = c(4);
  TRAINING = w1 w2 w3 w4 (BCH); MISSING = ALL (-999);
ANALYSIS: TYPE = MIXTURE; ESTIMATOR = MLR; STARTS = 0;
MODEL:
  %OVERALL%
  sosz2 ON sosz1 age sexf ses bmiz;
  sosz1 age sexf ses bmiz;
  %c#1%
  [sosz2] (m1);
  sosz2;
  %c#2%
  [sosz2] (m2);
  sosz2;
  %c#3%
  [sosz2] (m3);
  sosz2;
  %c#4%
  [sosz2] (m4);
  sosz2;
MODEL CONSTRAINT:
  NEW(d2 d3 d4);
  d2 = m2 - m1;
  d3 = m3 - m1;
  d4 = m4 - m1;
MODEL TEST:
  0 = m2 - m1;
  0 = m3 - m1;
  0 = m4 - m1;
OUTPUT: ;
```
